# 1-Year Prevalence and Factors Related to Injuries and Illnesses in Japanese Judo Collegiate Athletes

**DOI:** 10.3390/jfmk9030148

**Published:** 2024-08-28

**Authors:** Akira Kinoda, Aleksandra Mącznik, Takeshi Kimura, Yuki Muramoto, Yoshinori Katsumata, Kazuki Sato

**Affiliations:** Institute for Integrated Sports Medicine, Keio University School of Medicine, 35 Shinanomachi Shinjuku-ku, Tokyo 160-8582, Japan; akirakiak@gmail.com (A.K.); alexmacznik@gmail.com (A.M.); takeshikimura4@gmail.com (T.K.); yukimuramoto1019@gmail.com (Y.M.); goodcentury21@gmail.com (Y.K.)

**Keywords:** judo, injuries, martial arts, epidemiology, risk factors, prevention

## Abstract

Despite its rich history and widespread participation, the research surrounding injuries and illness in judo remains relatively limited compared to other sports. The primary aim of this research was to investigate injuries and illness within a previous year in Japanese collegiate judo athletes and analyze possible factors associated with these. This was a cross-sectional observational study using a web-based survey to collect data on the 1-year prevalence of injuries and illness. This study involved 564 judo athletes (67% males), aged between 18 and 25 years. Of these, 344 athletes (61%) reported one or more injuries within the previous year, and 49 reported illness (9%). The more judo experiences the athlete acquired, the less likely they were to sustain an injury (OR: 0.9; 95% CI: 0.56–1.10; *p*-value < 0.05) or illness (OR: 0.9; 95% CI: 0.81–1.00; *p*-value < 0.05). Support of an athletic trainer was associated with 1.7 times increased odds of sustaining an injury (95% CI: 1.19–2.49; *p*-value < 0.05). Athletes with obese BMI status (BMI > 30) had 3.1 times higher odds of becoming ill (95% CI: 1.41–6.95; *p*-value = 0.005), and athletes training more than 5 days per week had the odds of becoming ill increased by 5.1 times (95% CI: 1.11–23.21; *p*-value = 0.036). Judokas with fewer years of experience and with obese status should be targeted in efforts to prevent injury and illness. Moreover, the support of an athletic trainer and the impact of weekly training days should be targeted in research efforts.

## 1. Introduction

Originating from Japan in the late 19th century, judo has evolved into a globally recognized sport, featured prominently in international competitions such as the Olympic Games. While judo offers numerous benefits, it is not without its inherent risks [1]. Injuries are an unfortunate reality in any sport, and judo is no exception. The combination of intricate throwing techniques, joint locks, and immobilizations exposes athletes to a wide array of potential injury mechanisms. Additionally, judo is a sport contested in weight-specific classes, which adds to the complexities of managing athletes’ health through constant monitoring of the body weight and composition, and the potential of risky practices such as rapid weight loss just before the competition. These practices as well as the intimate proximity and inherent physical contact with opponents might increase the risk of illness among judokas.

Furthermore, the close-contact nature of judo, with its constantly evolving rules, necessitates ongoing research to address emerging injury and illness profiles. Injuries may influence athlete performance and longevity in sport and illnesses can determine athlete’s success, being crucial elements in optimal athlete development. Japanese judokas are regarded as some of the best in the world, and knowing their injury and illness profiles could be helpful in contextualizing that success. Understanding the prevalence, nature, and contributing factors of injuries and illness in judo is essential for the development of effective prevention strategies, as well as for optimizing performance and athlete well-being.

Despite its rich history and widespread participation, the research surrounding injuries in judo remains limited compared to other sports [2,3]. Available data are only irregular, resulting in none of the judokas’ populations being researched thoroughly. The available data span different nationalities, ages, and levels, from elite Brazilian [4] or club-level Mongolian youth [5] through French regional and national levels of all ages [3] to the international elite Olympic-level judokas [6,7,8,9,10]. Research in the collegiate population specifically is extremely scarce with only one study in Polish physical education students taking a judo class as a part of their curriculum [11]. Additionally, establishing whether more injuries happen to female or male judokas, or in training versus competition, could allow for immediate application of preventive strategies in athlete development at any stage.

Finally, the research on illnesses in judokas is limited with only three studies ever looking at this topic [6,9,10]. All three studies were carried out during the Olympics and only gave the total prevalence numbers without any details. The impact of the limited research on injury and illness prevalence in judo athletes is potentially disastrous with coaches and athletes basing their training and competition decisions on guesses.

The primary aim of this research was to investigate the injuries and illnesses within a previous year in Japanese collegiate judo athletes. Additionally, we aimed to analyze possible factors associated with higher odds of sustaining an injury or contracting an illness.

## 2. Materials and Methods

This was a cross-sectional observational study using a web-based retrospective survey to collect data on 1-year prevalence of injuries and illness in collegiate judo athletes. The study was designed and conducted in accordance with the Declaration of Helsinki [12] and reported according to the STROBE-SIIS consensus statement [13]. The Ethics Committee of Keio University approved this study (approval number: 20211158).

Collegiate judo athletes associated with Japan Association for University Athletics and Sport (UNIVAS) were eligible and recruited from June to October 2022. Informed consent was collected digitally before athletes could access the web-based survey. Data submitted by non-athletes were excluded from the analysis.

The survey was hosted on a specially designed website (https://enquete.cc/q/BC2XC8A8; accessed on 1 June 2022). The questions were adapted from the latest consensus document of the Japanese Society of Clinical Sports Medicine and the Japanese Society for Athletic Training [14] and adjusted to better fit the collegiate demographic.

The survey consisted of questions on athlete’s characteristics (age, sex, judo experience, competition level, practice days per week, number of tournaments in a season, hand and leg dominance, year at university, height, weight, body mass index (BMI), support from an athletic trainer–a person who takes care of the athletes on a day-to-day basis providing first aid, injury prevention, and management), injuries, and illnesses experienced within the previous year. For injuries, location, severity, type, time lost from training/competition, mechanism, and onset data were collected. Diagnosis, severity, time lost from training/competition, and onset data were collected for illnesses.

Injury was defined as any tissue damage or other derangement of normal physical function that occurred during sports participation [14]. Illness was defined as a complaint or disorder experienced by an athlete not related to injury [14]. The severity was derived from the time loss from training and competition as follows: minimal (0 days lost), mild (1 day–1 week lost), moderate (1 week–1 month lost), severe (1 month–6 months lost), and very severe (>6 months lost) [13,15].

The statistical software SPSS (IBM Corp, Armonk, NY, USA. Release 2021 version 28.0) was employed for conducting statistical analysis. Continuous data were summarized using mean and standard deviation. Discrete data were summarized by calculating counts and percentages. A *p*-value lower than 0.05 was deemed statistically significant. To determine the one-year period prevalence, the number of athletes who sustained injuries/fell ill was divided by the total number of athletes and multiplied by 100%. Differences in injury and illness characteristics, such as location, severity, type, onset, mechanism, and time lost between females and males, injured and uninjured, and athletes with illness and no illness were compared using the chi-square test (Pearson’s chi-square, Fisher’s exact test, or Fisher’s exact test with Monte Carlo estimates, as appropriate). To evaluate the factors associated with injury and illness occurrence, regression models incorporating participant demographics and sports participation were utilized. Logistic regression was employed to estimate odds ratios (ORs) with 95% confidence intervals for each outcome variable.

## 3. Results

### 3.1. Characteristics of Japanese Collegiate Judo Athletes

This study involved 564 judo athletes (67% males) aged between 18 and 25 years and with an average of 12 years of experience in the sport. The characteristics of the athletes are presented in Table 1. The athletes were mainly at a regional or national level, 84% competed between 1 and 5 times per season, and on average were training six days per week.

### 3.2. Injuries and Illnesses

Out of 564 athletes, 344 athletes (61%) reported 581 injuries within the previous year, 15 reported COVID-19, and 34 reported other illnesses (9%). The characteristics of injuries and illnesses are presented in Table 2.

The overwhelming majority of injuries involved limbs, specifically joints. Most of the injuries (86%) required some time off from training and competition. Males reported a higher proportion of head injuries, moderate injuries, recurrent injuries, injuries occurring in training, and due to the direct mechanism. Females reported a higher proportion of elbow and ACL injuries and new injuries. In terms of illness, the only difference in proportions between males and females was in severity, where males reported a higher proportion of mild illnesses than females.

COVID-19 was the most reported illness (31%), followed by digestive (27%) and skin issues (16%).

### 3.3. Differences between Injured and Uninjured Judo Athletes

Differences between injured and uninjured athletes in proportion to various characteristics are presented in Table 3.

Differences between injured and uninjured athletes were found in proportions of the year of study, and the presence or lack of support from an athletic trainer. No differences in sex, BMI, practice days per week, frequency, and level of competition were detected.

### 3.4. Differences in Characteristics of the Athletes (n = 564) Who Reported Illness or No Illness

Differences in characteristics’ proportions between athletes who became ill and did not become ill are presented in Table 4. Differences between athletes with illness and those without were found in BMI and competition level, but not in sex, year at university, BMI status, practice days per week, competition frequency, support, or health status.

### 3.5. Factors Related to Sustaining an Injury in Judo Athletes

An analysis of the factors related to sustaining an injury is presented in Table 5. Fewer years of experience in judo and support from an athletic trainer were the only factors related to increased odds of sustaining an injury.

### 3.6. Factors Related to Experiencing an Illness in Judo Athletes

The factors related to illness in judo athletes are presented in Table 6. Obese BMI status, fewer years of experience, and more than 5 practice days per week were associated with higher odds of falling ill in Japanese judo athletes. Factors such as age, sex, year at university, competition level, and frequency were not associated with illness.

## 4. Discussion

Years of judo experience and support from an athletic trainer were the factors influencing the odds of sustaining an injury and BMI status, years of judo experience, and practice days per week were the factors influencing the odds of contracting an illness in Japanese judo athletes. Having the support of an athletic trainer was associated with 1.7 times increased odds of sustaining an injury. Athletes with obese BMI status (BMI > 30) had 3.1 times higher odds, and athletes practicing judo more than five days per week had 5.1 times higher odds of becoming ill. The more experience the judo athletes acquired in the sport, the less likely they were to both sustain an injury or fall ill. Factors such as age, sex, year at the university, frequency of competition, and competition level did not influence the odds of injury or illness in this population.

### 4.1. Prevalence of Injuries and Illness

Among Japanese collegiate judo athletes, 61% had been injured and 9% had become ill within the previous year. The injury prevalence found in this study seems similar to the other studies reporting a 10-year prevalence of 64.8% for musculoskeletal injuries in Brazilian state-associated judokas [16], but higher than ‘current injury’ reported by 39% of Mongolian judo club athletes (aged 9–22) [5] and 13.5% of French judokas injured within three competitions [17], 10–15% during Olympic tournaments [6,7,9,10], and 1.1% injured in the 21-year-long study of judo competitions in France [3].

In general, we found that injury patterns are very similar in male and female judo athletes, as did other studies [18]. However, slight differences exist. For example, a higher proportion of male athletes experienced head injuries, injuries of moderate severity, in season, in training, and due to the direct mechanism when compared to their female counterparts. Females experienced a higher proportion of elbow and ACL injuries than males. Mild illness and in-season illness were experienced in a higher proportion by males than females.

Moreover, this study found 2.6 times more injuries reported in training than in the competition, with 60% of injuries being sustained to the lower limbs. Lower-limb injuries (44%) were also most common in the 4-year investigation of national-level Korean athletes [18]. However, a study on competition injuries in French judokas showed that most of the injuries were to the upper body [3], a similar result to the results in three consecutive Olympics [8]. Therefore, perhaps the injuries sustained during training versus during the competition vary and should be investigated separately in future studies.

The illness prevalence in this study (9%) is high compared to the three available studies in judo Olympians of 4.2% in London [6], almost 3% in Rio [9], and almost 4% in Tokyo [10]. However, our study investigated 1-year prevalence in judo training and competition, whereas the other studies looked only at the prevalence during a judo Olympic tournament. Due to the fact that the timeframe, athlete level, and exposure type differed, these results should be considered with caution and within their respective contexts.

Illness prevalence in other sports showed however similar prevalence to the one found in this study. During multiday competition events, 6.8% of ill athletes were reported at the IAAF 2009 [19] and 2011 events [20], 7.2% at the 2010 Olympics [21], 8.2% at the 2012 Youth Olympics [22], 8.9% at the 2014 Olympics [23], or 12.1% at the FIFA 2010 [24]. Moreover, another study investigating illness prevalence in female high school students found that only 7.2% of athletes became ill but as much as 27.6% of non-athletes reported illness [25]. The illness prevalence in this study is around the prevalence reported for other sports but higher than in judo-specific studies, warranting more research.

### 4.2. Factors Associated with Injury

Sports experience had an injury-protective effect on judo athletes surveyed in this study. Other studies have reported contrary results with age or experience being factors promoting injuries. More years of experience were associated with higher odds of sustaining both acute and overuse injuries in the Finnish population (various sports) [26]. In judo collegiate athletes, age was associated with higher odds ratios of injuries [11]. The athletes, however, were of recreational status and chose judo as their PE subject rather than being devoted specialists. In a 21-year-long surveillance study in French judokas, the ones who competed at the national level experienced more injuries (1.44%) than regional and district judokas (0.9%) [3]. Therefore, a possible explanation for the findings of this study would be that more experienced athletes had more time to train their bodies to adapt to training and competitions. Longer training time also could have allowed for the accumulation of technical practice necessary to counter the attacks of the opponents or master advanced techniques of safe falls, and therefore reduce the likelihood of sustaining an injury. As judo is a combat sport where the opponents are actively trying to cause physical harm to each other, experience in defense techniques is crucial. Other aspects influencing this outcome could include accumulated training load, fitness levels, and competition experience as suggested for combat sports in general [8].

Support of an athletic trainer was associated with higher odds of sustaining an injury in collegiate Japanese judo athletes. This finding contradicts other studies that reported athletic trainers reducing rates and recurrence of injuries and proving to be effective in recognizing concussions in the US high school population [27]. Support from athletic trainers was reported by the injured US collegiate athletes to foster belief in successful recovery, normalize the struggle of the rehabilitation process, and help set clear goals and expectations [28]. Moreover, athletic trainer services were suggested as a cost-effective healthcare modality in school and community-based sports [29,30].

The influence of athletic trainers on increased injury prevalence found in this study may be due to several aspects. Athletes who had access to an athletic trainer could have increased their training volume and/or intensity due to treatment or advice given. Also, treatment such as taping or fitting with braces could have kept athletes with minimal injuries in play/competition, perhaps causing more severe injury in the long run. On the other hand, athletic trainers could have kept the athletes longer in rehabilitation, thoroughly checking before allowing for a return to play, and therefore causing the reported injuries to appear more serious when compared to the injuries sustained by athletes without access to an athletic trainer. Finally, athletic trainers’ care and education could have allowed the athletes to report the injuries more accurately. Additional attention from athletic trainers could have made an athlete remember the injury better and ensure improved recall in the retrospective survey.

### 4.3. Factors Associated with Illness

Years of experience in judo have been shown to protect against illness in Japanese collegiate athletes. Similarly, one study showed that younger collegiate female students were more likely to contact COVID-19 [31]. This is an interesting finding as sports have been shown to expose athletes to multiple stressors with the potential to depress immunity and increase the risk of illness [32]. Our findings, however, indicate that as the athletes move into later years in the sport, they probably learn how to navigate these stressors better to mitigate the risks of falling ill [33,34,35].

Obese BMI status was associated with higher odds of contracting illness in judo athletes. Athletes carrying more fat tissue may be more susceptible to hormonal and immunological changes that predispose them to illness. The literature on BMI and illness in athletes is limited. However, one study [31] showed that athletes who contracted COVID-19 had higher BMI than the ones who had not. There are studies showing obese status as a protective factor against sports injury in Canadian adolescent athletes [36], and other studies looking at child, youth, and adolescent populations show an overall negative effect of high BMI on sports injuries ranging in an increase of odds from 1.4 to 3.9 [37]. Bringing all the findings together may mean that although obese BMI status is not a detrimental factor for a sports injury in children, it may become one later in their career. Children develop fast and periodically experience growth spurts. Having a reserve of fat tissue to secure energy supply during the development may have a positive effect. As the athletes mature, however, the requirements of carrying a heavier body may exceed benefits and predispose the athletes to injuries.

Practicing judo more than five days per week was associated with 5.1 times higher odds of becoming ill. This outcome could be related to the increased possibility of overtraining and/or under-recovery, which were both indicated as factors driving immunodepression [25]. Therefore, research into the impact of training load on illness in judo or sports in general is warranted.

### 4.4. Limitations

This study presents several limitations that need consideration. The retrospective design of data collection in this study could have introduced a retrieval bias, where athletes could not quite remember actual timeframes for their injury and/or illness. Athletes’ injuries or illnesses were self-reported which could increase the number of reported cases. Additionally, we asked for details for up to the three most serious injuries, which could have resulted in underreporting. However, considering the retrospective design of this study, asking about the three most serious injuries aimed at assuring high-quality data, as the athletes are more likely to remember the details of these injuries. Moreover, data collected on injury details were limited, and therefore no injuries could be proposed for specific injury risk reduction efforts. The sample of athletes included in this study was homogenous, consisting only of Japanese collegiate judo athletes. Therefore, the results obtained may not be generalizable to the global population or other age/sports groups.

## 5. Conclusions

Judo athletes who are less experienced and have the support of an athletic trainer have higher odds of sustaining an injury. Judokas with less experience, obese BMI status, and training more than five days per week have higher odds of contracting illness.

The findings of this study suggest that less experienced judokas should be targeted with injury and illness prevention strategies. The role of athletic trainers in support of judo athletes should be investigated more thoroughly for tangible recommendations. Athletes with obese BMI status may be more susceptible to contracting an illness and therefore preventive measures, specifically for respiratory and skin diseases, should be undertaken. Special attention should be given to the training load the judokas are undertaking, as these could influence their susceptibility to illness.

Ultimately, the findings of this research paper will contribute to a deeper understanding of injuries and illnesses related to judo participation, fostering a safer environment for judo practitioners of all levels. Through informed interventions, such as rule modifications, athlete education, and improved training protocols, we can strive to minimize the frequency and severity of injuries, thereby enhancing the overall experience and longevity of judo participants.

## Figures and Tables

**Table 1:** Characteristics of judo collegiate athletes (n = 564).

Characteristic	All n = 564 100%	Males n = 376 66.7%	Females n = 183 32.4%	Sex Undisclosed n = 5 0.5%
Age, years; mean ± SD	19.8 ± 1.2	19.8 ± 1.2	19.9 ± 1.3	20.4 ± 1.1
Year at university; n (%)				
Year 1	183 (32.4)	122 (32.4)	61 (33.3)	0 (0)
Year 2	138 (24.5)	100 (26.6)	37 (20.2)	1 (20.0)
Year 3	142 (25.2)	89 (23.7)	50 (27.3)	3 (60.0)
Year 4+	101 (17.9)	65 (17.3)	35 (19.1)	1 (20.0)
Height, cm; mean ± SD	167.7 ± 8.2	171.6 ± 6.1	159.5 ± 5.6	168.4 ± 9.7
Weight, kg; mean ± SD	76.5 ± 18.8	82.8 ± 18.3	63.5 ± 11.7	73.5 ± 24.6
BMI *, kg/m^2^; mean ± SD	27.0 ± 5.1	28.0 ± 5.3	24.9 ± 4.1	25.6 ± 6.8
Judo experience, years; mean ± SD	12.2 ± 3.7	12.2 ± 3.7	12.3 ± 3.8	9.8 ± 1.3
Competition level; n (%)				
regional	248 (44.0)	188 (50.0)	57 (31.1)	3 (60.0)
national	301 (53.4)	183 (48.7)	116 (63.4)	2 (40.0)
international	15 (2.6)	5 (1.3)	10 (5.5)	0 (0)
Practice days per week; mean ± SD	5.7 ± 0.9	5.7 ± 0.8	5.7 ± 1.0	5.0 ± 2.2
Tournaments per season; n (%)				
1–5 matches	473 (83.9)	316 (84.0)	152 (83.1)	5 (100.0)
6–10 matches	77 (13.7)	53 (14.1)	24 (13.1)	0 (0)
11–15 matches	10 (1.8)	4 (1.1)	6 (3.3)	0 (0)
>16 matches	4 (0.7)	3 (0.8)	1 (0.5)	0 (0)
Hand dominance; n (%)				
right	474 (84.0)	308 (81.9)	161 (88.0)	5 (100)
left	78 (13.8)	57 (15.2)	21 (11.5)	0 (0)
both	12 (2.1)	11 (2.9)	1 (0.5)	0 (0)
Leg dominance; n (%)				
right	458 (81.2)	302 (80.3)	153 (83.6)	3 (60.0)
left	98 (17.4)	68 (18.1)	2 (15.3)	2 (40.0)
both	7 (1.2)	6 (1.6)	1 (0.5)	0 (0)
Reported health; n				
injury	344	225	118	1
illness	49	31	18	0
none (no injury and no illness)	202	139	59	4
both (injury and illness)	31	19	12	0
Injuries per year; n (%)				
none	220 (39.0)	151 (40.2)	65 (35.5)	4 (80.0)
one	179 (31.7)	115 (30.6)	64 (35.0)	0 (0)
two	100 (17.7)	63 (16.8)	37 (20.2)	0 (0)
three or more	65 (11.5)	47 (12.5)	17 (9.3)	1 (20.0)
Illness; n (%)				
COVID-19	15 (30.6)	10 (32.3)	5 (27.8)	0
other illness	34 (69.4)	21 (67.7)	13 (72.2)	0
Support *; n (%)				
AT	165 (29.3)	112 (29.8)	51 (27.9)	2 (40.0)
Non-AT	399 (70.7)	264 (70.2)	132 (72.1)	3 (60.0)

* BMI—body mass index; AT—had the support of an athletic trainer, Non-AT—had no support of an athletic trainer.

**Table 2:** Characteristics of injuries (n = 581) and illnesses (n = 49) in judo collegiate athletes (n = 564).

Characteristic	All	Males	Females	Difference in Proportions between Males and Females	Sex Undisclosed
				[Pearson’s χ^2^]	
				*p*-value	
INJURY	581 *	388	190		3
Location; n (%)					
Head	23 (4.0)	**20**	3	**0.039**	0
head	14	12	2	0.134	0
face	9	8	1	0.206	0
Trunk	61 (10.5)	42	18	0.617	1
cervical spine	9	6	3	0.976	0
chest	10	7	3	0.845	0
upper back	5	5	0	0.116	0
abdomen	2	2	0	0.322	0
lumbo-sacral spine	30	21	9	0.731	0
buttock	4	1	3	0.072	0
groin	1	0	0	NA *	1
Upper limb	195 (33.6)	123	71	0.175	1
shoulder	71	50	21	0.528	0
upper arm	0	0	0	NA *	0
elbow	60	33	**27**	**0.035**	0
lower arm	1	1	0	0.484	0
wrist	18	9	8	0.206	1
hand	13	8	5	0.665	0
finger	29	20	9	0.829	0
thumb	3	2	1	0.987	0
Lower limb	302 (52.0)	203	98	0.867	1
hip joint	1	0	1	0.153	0
thigh	18	10	8	0.288	0
knee	121	88	33	0.140	0
ACL *	30	14	**16**	**0.014**	0
Achilles	0	0	0	NA*	0
lower leg	9	7	2	0.493	0
ankle	41	29	11	0.454	1
toe	35	20	15	0.195	0
fracture	47	35	12	0.264	0
	Missing 14	Missing 12	Missing 2		Missing 0
Time lost; n (%)					
yes	500 (86.1)	339 (86.9)	161 (84.3)	0.142	2
no	81 (13.9)	51 (13.1)	30 (15.7)	0.142	1
Severity; n					
minimal (0 days lost)	77	45	32	0.795	1
mild (1 day–1 week lost)	115	73	42	0.396	1
moderate (1 week–1 month lost)	226	**166**	60	**<0.001**	0
severe (1 month–6 months lost)	124	83	41	0.084	1
very severe (>6 months lost)	39	23	16	0.911	0
Type; n					
new	407	272	**135**	**<0.001**	2
recurrent	158	**109**	49	**0.010**	0
overuse	16	9	7	0.768	1
Onset (part of the season); n					
pre-season	196	117	79	0.964	3
in season	327	**238**	89	**<0.001**	0
post-season	44	27	17	0.832	0
other	14	8	6	0.836	0
Onset (competition or training); n					
in competition	160	101	59	0.353	0
in training	421	**289**	132	**<0.001**	3
Mechanism; n					
direct	398	**275**	123	**<0.001**	3
indirect	118	72	46	0.780	0
non-contact	65	43	22	0.282	0
ILLNESS	49	31	18		
Diagnosis, n					
COVID-19	15	10	5	0.743	0
digestive issues	13	6	7	0.135	0
skin issues	8	5	3	0.961	0
heat stroke	5	4	1	0.413	0
hay fever	3	3	0	0.173	0
urological issues	3	3	0	0.173	0
gynecological issues	2	0	2	0.058	0
Time lost; n					
yes	47	30	17	0.691	0
no	2	1	1	0.691	0
Severity; n					
minimal (0 days lost)	2	1	1	NA *	0
mild (1 day–1 week lost)	10	**8**	2	**0.016**	0
moderate (1 week–1 month lost)	25	17	8	0.170	0
severe (1 month–6 months lost)	8	5	3	0.187	0
very severe (>6 months lost)	4	0	4	**0.004**	0
Onset (part of the season); n					
pre-season	13	6	7	0.612	0
in season	25	17	8	0.170	0
post-season	11	7	4	0.201	0

* each athlete could report up to three injuries; ACL—anterior cruciate ligament; NA—not applicable.

**Table 3:** Differences between injured and uninjured judo athletes (n = 564).

Characteristic	Injured	Uninjured		
	n = 344	n = 220	χ^2^	*p*-Value
Sex; n				
male	225	151	4.677	0.096
female	118	65		
undisclosed	1	4		
Year at university; n (%)				
Year 1	85 (24.7)	98 (44.5)	24.563	**<0.001**
Year 2	91 (26.5)	47 (21.4)		
Year 3	97 (28.2)	45 (20.5)		
Year 4+	71 (20.6)	30 (13.6)		
BMI; n (%)				
underweight + normal 18.5–24.9	152 (44.2)	94 (42.7)	1.660	0.436
overweight 25.0–29.9	123 (35.8)	72 (32.7)		
obese > 30.0	69 (20.0)	54 (24.6)		
Competition level; n				
regional	145	103	1.245	0.537
national	190	111		
international	9	6		
Practice days per week; n				
1	1	6	12.146	0.059
2	2	5		
3	5	3		
4	11	9		
5	33	14		
6	284	180		
7	8	3		
Tournaments per season; n				
1–5	284	189	2.604	0.626
6–10	49	28		
11–15	8	2		
16–20	2	1		
21–25	0	0		
26–30	1	0		
>31	0	0		
Support *; n (%)				
AT	113 (32.8)	52 (23.6)	5.502	**0.023**
Non-AT	231 (67.2)	168 (76.4)		

* AT—had the support of an athletic trainer, Non-AT—had no support of an athletic trainer.

**Table 4:** Differences in characteristics of the athletes (n = 564) who reported illness (n = 49) versus no illness (n = 515).

Characteristic	Illness	No Illness		
	n = 49	n = 515	χ^2^	*p*-Value
Sex; n				
male	31	345	0.873	0.646
female	18	165		
undisclosed	0	5		
Year at university; n				
Year 1	13	170	0.994	0.803
Year 2	14	124		
Year 3	13	129		
Year 4+	9	92		
BMI; n (%)				
underweight BMI < 18.4 + Normal BMI 18.5–24.9	15 (6.1)	231 (93.9)	9.408	**0.009**
overweight BMI 25.0–29.9	15 (7.7)	180 (92.3)		
obese BMI > 30.0	19 (15.4)	104 (84.6)		
Practice days per week; n				
1	1	6	8.365	0.213
2	0	7		
3	0	8		
4	1	19		
5	0	47		
6	45	419		
7	2	9		
Tournaments per season; n				
1–5	41	432	0.418	0.981
6–10	7	70		
11–15	1	9		
16–20	0	3		
21–25	0	0		
26–30	0	1		
>31	0	0		
Support *; n				
AT	19	146	2.350	0.088
Non-AT	30	369		
Competition level; n (%)				
regional	22 (8.9)	226 (91.1)	6.538	**0.038**
national	23 (7.6)	278 (92.4)		
international	4 (26.7)	11 (73.3)		
Health status; n				
injury	31	313	0.116	0.762
no-injury	18	202		

* AT—had the support of an athletic trainer, Non-AT—had no support of an athletic trainer.

**Table 5:** Factors related to sustaining an injury in judo athletes.

Factor	Odds Ratio	95% CI	*p*-Value
Age	0.786	0.560–1.102	0.162
Sex			
male	reference		
female	0.791	0.524–1.193	0.263
Year at university			
Year 1	reference		
Year 2	0.579	0.320–1.048	0.071
Year 3	0.784	0.342–1.796	0.565
Year 4+	0.963	0.298–3.113	0.963
Judo experience	**0.913**	**0.560–1.102**	**<0.05**
BMI			
normal + underweight < 25	reference		
overweight 25.0–30.0	1.109	0.677–1.817	0.580
obese > 30.0	0.885	0.574–1.364	0.681
Support *; n (%)			
Non-AT	reference		
AT	**1.656**	**1.101–2.489**	**<0.05**
Competition level			
regional	reference		
national	1.196	0.843–1.695	0.315
international	0.943	0.317–2.802	0.916
Practice days per week			
1–5	reference		
6–7	0.902	0.548–1.483	0.684
Tournaments per season;			
1–5 matches	reference		
>6 matches	1.078	0.638–1.821	0.779

* AT—had the support of an athletic trainer, Non-AT—had no support of an athletic trainer.

**Table 6:** Factors related to illness in judo athletes.

Factor	Odds Ratio	95% CI	*p*-Value
Age	0.604	0.337–1.083	0.091
Sex			
male	reference		
female	1.604	0.778–3.308	0.201
Year at university			
Year 1	reference		
Year 2	1.804	0.750–4.338	0.187
Year 3	1.741	0.698–4.341	0.235
Year 4+	2.249	0.805–6.281	0.122
Judo experience	**0.899**	**0.810–0.998**	**0.046**
BMI			
normal + underweight	reference		
overweight 25.0–30.0	1.130	0.488–2.615	0.776
obese > 30.0	**3.134**	**1.413–6.951**	**0.005**
Support *; n (%)			
Non-AT	reference		
AT	1.447	0.749–2.797	0.272
Competition level			
regional	reference		
national	0.854	1.012–16.856	0.663
international	3.196	0.794–12.858	0.102
Practice days per week			
1–5	reference		
6–7	**5.073**	**1.109–23.211**	**0.036**
Tournaments per season;			
1–5 matches	reference		
>6 matches	1.065	0.464–2.449	0.881

* AT—had the support of an athletic trainer, Non-AT—had no support of an athletic trainer.

## Data Availability

All data from these studies are contained within this manuscript or are available from the corresponding author upon reasonable request.
